# Decision-Making Based on Social Conventional Rules by Elderly People

**DOI:** 10.3389/fpsyg.2018.01412

**Published:** 2018-08-13

**Authors:** Hidetsugu Komeda, Yoko Eguchi, Takashi Kusumi, Yuka Kato, Jin Narumoto, Masaru Mimura

**Affiliations:** ^1^Department of Education, College of Education, Psychology and Human Studies, Aoyama Gakuin University, Tokyo, Japan; ^2^Department of Neuropsychiatry, Keio University School of Medicine, Tokyo, Japan; ^3^Department of Cognitive Psychology in Education, Graduate School of Education, Kyoto University, Kyoto, Japan; ^4^Department of Psychiatry, Graduate School of Medical Science, Kyoto Prefectural University of Medicine, Kyoto, Japan

**Keywords:** elderly, social decision, good–bad judgments, behavior, trust, fraud, narrative comprehension

## Abstract

Information used by older adults engaging in a social decision making task of judging a protagonist as a good or a bad person was investigated. Older (*n* = 100, 50 women, mean age = 63.6 years) and younger (*n* = 100, 50 women, mean age = 25.7 years) adults participated in a web-based survey. In Experiment 1, we assessed participants’ rapid decision-making processes when making good or bad judgments after reading consecutive sentences without reviewing previously read sentences. The percentages of good judgments were analyzed. In Experiment 2, two protagonists engaging in a deliberate decision-making process were presented, and participants were asked to judge better and worse protagonists. The percentages of behavior-based judgments were analyzed. Results of Experiment 1 indicated that older adults judged protagonists as “good” more often than younger adults. Especially, older adults judged protagonists with good behavior as being “good.” In Experiment 2, older adults made behavior-based judgments more than younger people. Additionally, older and younger adults used information on personalities of protagonists for making judgments in situations with bad outcomes, or incongruent. Moreover, multiple regression analysis suggested that people with more general trust engaged more, whereas people with more caution engaged less in making behavior-based judgments.

## Introduction

Japan is confronting a rapidly-aging society. In 2015, the aging rate of the population (the ratio of people that are 65 years or older compared to the total population) was 26.8%. This figure is estimated to reach approximately 30% and 40% in 2025 and 2060 respectively (National Institute of Population and Social Security Research, 2012). Criminal activities targeting the elderly have increased with the increase in the aging rate of the population. This is especially true of bank transfer frauds in which an estimated 86.1% of the victims are at least 65 years old (Cabinet Office, Government of Japan, 2014).

There are several reasons why older adults are more likely to become victims of fraud. First, older adults have an increased risk of dementia. Especially, it is known that older adults with limited literacy are at an increased risk for dementia (Kaup et al., 2014). Second, older adults are known to show a favorable bias toward people that are visually perceived as trustworthy over those that look untrustworthy, which persists even after older adults have been cheated by trustworthy-looking people as often as by untrustworthy-looking ones (Suzuki, 2018). This suggests that older adults are less likely than younger adults to learn to avoid face-based trustworthiness judgments. Taken together, this decision-making processes may vary as a function of aging. This study was designed to investigate interpersonal decision-making processes to clarify the reasons that make older adults become fraud victims.

Interpersonal decision making has been shown to involve at least three possible components: Trait inferences based on others’ characteristics (Bargh et al., 1996; Todorov et al., 2005; Suzuki, 2018), inferences based on behaviors (Betancourt, 1990), and evaluation of outcomes as outputs (Peters et al., 2000). Also, older adults are known to be more likely to make stereotypical inferences than younger adults, causing them to be more prejudiced than younger adults (Radvansky et al., 2009, 2010; Narvaez et al., 2011; Sato, 2013).

Moreover, older adults are more likely to make inferences based on conventional social rules when reading moral stories than younger adults (Haidt, 2003). It is unclear whether the characteristics of a protagonist, such as “Yoko-san is kind to her mother,” or behavioral information, such as “She tasted the sweet azuki (sweet red-bean) soup and gave it to her mother,” are used by older adults in their interpersonal decision-making processes of narrative comprehension. Therefore, this study focused on “good” and “bad” judgments regarding narrative story protagonists.

People tend to invent post-hoc reasons for conflicting intuitions that arise in their daily experience (Nisbett and Wilson, 1977; Haidt, 2003). Therefore, the present study examined the effect of the congruency or incongruence between traits (as a characteristic) and behaviors on decision making, as well as the separate effects of traits and behavioral inferences, by creating discrepancies in information that resembled situations of social conflict. We developed novel stories based on stories for typically and atypically developing adolescents (Komeda et al., 2016), in which the protagonist’s characteristics and behaviors, as well as outcomes, were manipulated. There have been numerous studies examining the influence of aging on situation model construction (Johnson-Laird, 1983; van Dijk and Kintsch, 1983; Zwaan and Radvansky, 1998), which is a reader’s mental representation of a fictional story based on textual representations as well as previous knowledge or experience. These studies have suggested that older adults show a decline in certain levels of processing, such as surface form and text-based levels. However, situation model level processing is relatively well preserved (Radvansky, 1999; Radvansky et al., 2003; Stine-Morrow et al., 2004; Radvansky and Dijkstra, 2007). Therefore, we selected story materials to construct situation model in order to assess interpersonal decision-making processes in aging.

The aim of this study was to examine the interpersonal social decision-making information used by older adults when judging a story protagonist as good or bad. Social decision making is defined as decision making about social interactions in complex situations (Rilling and Sanfey, 2011). In Experiment 1, we assessed participants’ rapid decision-making processes when making good-bad judgments after reading consecutive sentences without reviewing previously read sentences. In Experiment 2, two protagonists engaging in a deliberate decision-making process were visually presented and participants were asked to judge the better and the worse protagonist. We predicted that older adults would judge protagonists using behavioral information during the social decision-making processes because they would be more likely than younger adults to make appropriate inferences about the behavior of others (Happé et al., 1998). More specifically, older adults were expected to make stereotypical decisions during rapid decision making (Radvansky et al., 2009, 2010) Therefore, we predicted that older adults would make appropriate decisions in the deliberate decision processes.

## Experiment 1

### Method

#### Participants

It is difficult to collect a large sample of older adults comprising several age ranges from a single community. Therefore, we used Cross Marketing, an online survey company. Participants registered with this company respond to research surveys for compensation. We recruited 100 older adults (50 women and 50 men, mean age = 63.6 years, range: 60–69 years old) and 100 younger adults (50 women and 50 men, mean age = 25.7 years, range: 22–29 years of age). All 200 participants were selected to have exactly 16 years of education leading to a university degree to ensure no differences in years of schooling between older and younger groups. Therefore, the differences between younger and older adults in the current study could not be explained by the years of schooling.

#### Stimuli and Procedure

As shown in **Table 1**, a story consisted of three sentences (first sentence: characteristics; second sentence: behavior; and third sentence: outcomes). The number of letters in the third sentences was identical across all stories. The stories were presented on a screen, one sentence at a time. Each sentence remained on the screen until the participant pressed a key, when the next sentence appeared. Participants could not refer back to previous sentences but could read each presented sentence at their own pace. They completed the survey at home or in a quiet place through the internet.

**Table 1:** Sample stories in Experiment 1.

Sample stories with good characteristics and good outcomes inExperiment 1.	
*Good characteristics*	*Good characteristics*
*with Good behavior*	*with Bad behavior*

Yoko-san is kind to her mother.	Yoko-san is kind to her mother.

She tasted the sweet azuki (red-bean)	She tasted the sweet azuki (red-bean)
soup and gave it to her mother because	soup and gave it to her mother even
it was delicious.	though it tasted bad.

She was glad to see that her mother was eating her favorite sweets.	

	
**Sample stories with bad characteristics and bad outcome in Experiment 1.**	

*Bad characteristics*	*Bad characteristics*
*with Good behavior*	*with Bad behavior*

Kenta-san is a noisy neighbor.	Kenta-san is a noisy neighbor.

He teaches his father how to use the	He makes purchases using his father’s
Internet.	credit card.

He is sad because his father suspects him of misdeeds.	


After reading each story, participants judged its protagonists as either good or bad. They read 24 stories, three stories for each combination of two characteristics (good, bad) × 2 behaviors (good, bad) × 2 outcomes (good, bad). The participants responded to two practice trials before the experimental trials to familiarize themselves with the reading procedure that was based on our previous study (Komeda et al., 2016).

### Results

#### Analyses of Good Judgments

**FIGURE 1:**
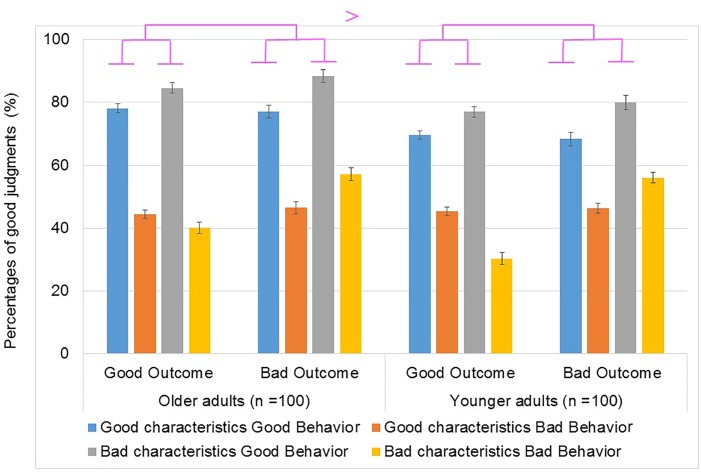
Percentages of “good” judgments in Experiment 1. The blue bar shows good characteristics with good behaviors, the red dark orange bar shows good characteristics with bad behaviors, the green gray bar shows bad characteristics with good behaviors, and the purple light orange bar shows bad characteristics with bad behaviors. Error bars represent 95% confidence intervals.

We next conducted a group × characteristics × behaviors × outcomes analysis of variance (ANOVA) on the percentages of good judgments (**Figure 1**). Results of the ANOVA indicated that the main effect of group was significant, *F* (1,198) = 7.36, *p* = 0.00, $\eta_{p}^{2}$ = 0.04. Older participants (*M* = 64.9%) judged the protagonists in the stories as “good” more often than younger participants (*M* = 58.8%). Moreover, the interaction between group × behavior of judgments was significant [*F*(1,198) = 5.56, *p* = 0.03, $\eta_{p}^{2}$ = 0.02]. A simple effects test revealed that protagonists with good behaviors were judged as “good” more often than protagonists with bad behaviors by both the older [*M* = 82.4% for good behavior, *M* = 47.4% for bad behavior; *F*(1,99) = 556.17, *p* < 0.001, $\eta_{p}^{2}$ = 0.85] and the younger group [*M* = 73.5% for good behavior, *M* = 44.1% for bad behavior; *F*(1,99) = 254.40, *p* < 0.001, $\eta_{p}^{2}$ = 0.72]. Interestingly, protagonists with good behaviors were judged by the older group (*M* = 82.4%) as “good” more often than by the younger group [*M* = 73.5%; *F*(1,198) = 15.47, *p* = 0.00, $\eta_{p}^{2}$ = 0.07]. However, there were no significant group differences in judging protagonists with bad behaviors [*M* = 47.4% by older, *M* = 44.1% or younger adults; *F*(1,198) = 1.41, *p* > 0.05, $\eta_{p}^{2}$ = 0.00].

The three-way interaction between group × outcome × behavior on judgments was also significant [*F*(1,198) = 3.92, *p* < 0.05, $\eta_{p}^{2}$ = 0.02]. A simple effects test indicated that the older group judged protagonists with good behaviors as “good” more often than protagonists with bad behaviors for good [*M* = 81.0% for good behaviors with good outcomes, *M* = 42.5% for bad behaviors with good outcomes, *F*(1,99) = 390.19, *p* < 0.001, $\eta_{p}^{2}$ = 0.80] and bad outcomes [*M* = 83.8% for good behaviors with bad outcomes, *M* = 52.3% for bad behaviors with bad outcomes; *F*(1,99) = 260.43, *p* < 0.001, $\eta_{p}^{2}$ = 0.72]. Similarly, the younger group judged protagonists with good behaviors as “good” more often than protagonists with bad behaviors for good [*M* = 73.3% for good behaviors with good outcomes, *M* = 36.7% for bad behaviors with good outcomes; *F*(1,99) = 246.99, *p* < 0.001, $\eta_{p}^{2}$ = 0.71] and bad outcomes [*M* = 73.7% for good behaviors with bad outcomes, *M* = 51.5% for bad behaviors with bad outcomes; *F*(1,99) = 92.12, *p* < 0.001, $\eta_{p}^{2}$ = 0.48].

### Discussion

Older participants judged protagonists as “good” more often than younger participants. This finding suggests that older people compared to younger people attended to more positive than negative information (Löckenhoff and Carstensen, 2007). This positivity effect is also evident in social decision making, for example, older people pay more attention to positive than to negative attributes when choosing doctors and hospitals (Löckenhoff and Carstensen, 2007, 2008), cars (Mather et al., 2005), and consumer products (Kim et al., 2008) more often than younger people. In other words, older adults show a preference for ignoring negative information and tend to rely on positive information more often than younger adults (Mather and Carstensen, 2005; Martins and Mather, 2016). Additionally, older adults try to find positive meanings in social relationships, even in situations of conflict (Brose et al., 2015). In this study also, older people attended to positive aspects of protagonists more than younger people when making social judgments.

Older adults judged protagonists with good behaviors as “good” more often than younger adults, suggesting that they judged protagonists based on behavioral information more than younger adults. However, this pattern was observed only for good behaviors and not for bad behaviors. Previous studies have shown that older adults engage in more conciliatory behaviors when reacting to interpersonal conflicts than younger adults (Birditt and Fingerman, 2005; Blanchard-Fields et al., 2007; Riediger and Luong, 2016). We first predicted that older adults would judge protagonists based on behavioral information. However, the results of the study indicated that older adults only used behavioral information for protagonists with good behaviors. Therefore, our prediction was only partially supported.

## Experiment 2

In Experiment 1, we assessed participants’ rapid decision-making processes when making good–bad judgments after reading consecutive sentences without reviewing previously read sentences. This paradigm was useful for investigating the processes of integration when making ongoing judgments. However, the accessibility to previous information was not controlled in Experiment 1: the second sentence describing behavioral information was closer to the outcome than the first sentence describing characteristics of the protagonists (Komeda et al., 2016). Additionally, this paradigm may have disadvantaged the older group, because they were required to remember three sentences when making their judgments.

In Experiment 2, all the sentences (in the two stories) were presented simultaneously to control for the accessibility of information. This enabled participants to compare different types of stories when both characteristics and behaviors of a protagonist were simultaneously available. As a result, we could identify the information that participants used for decision-making.

### Method

#### Participants

All participants that completed Experiment 1 also completed Experiment 2.

#### Stimuli and Procedure

**FIGURE 2:**
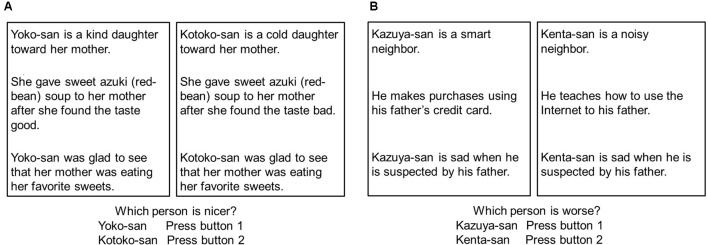
**(A)** Comparison task in good outcome condition in Experiment 2. The good outcome condition asked which protagonists were better. “-san” is an honorific suffix added to an adult’s name. **(B)** Comparison task in the bad outcome in Experiment 2. The bad outcome condition asked which protagonists were worse. “-san” is an honorific suffix added to an adult’s name.

As shown in **Figures 2A,B**, each outcome had two prior contexts. The gender and position (left or right presentation location) of the protagonists were counterbalanced similar to our previous study (Komeda et al., 2016). For good outcomes, good characteristics/good behavior was compared with bad characteristics/bad behavior (**Figure 2A**), and good characteristics/bad behavior was compared with bad characteristics/good behavior. In bad outcomes, bad characteristics/bad behavior was compared with good characteristics/good behavior, and bad characteristics/good behavior was compared with good characteristics/bad behavior (**Figure 2B**). In the case of good outcomes, participants were asked to judge which protagonist was better, and in the case of bad outcomes, they judged which protagonist was worse. Participants could rely on characteristics or behaviors for making their decisions. They were not instructed which information to use because we wanted to assess strategic differences across the groups.

After completing the comparison task (**Figures 2A,B**), all the participants completed the Trust Scale (Yamagishi and Yamagishi, 1994): This is a 5-point scale designed to assesses general trust (6 items, Range = 5–30, high score means high trust) and caution (7 items, Range = 7–35, high score means high cautious). In this scale, items of general trust are statements concerning honesty and trustworthiness of people in general, such as “Most people are basically honest,” “Most people are basically good and kind,” “Most people will respond in kind when they are trusted by others.” On the other hand, items on caution are statements that point out risks in social life and advise caution in dealing with others, such as “One can avoid falling into trouble by assuming that all people have a vicious streak,” “There are many hypocrites in this society,” “No matter what they say, most people inwardly dislike putting themselves out to help others” (Yamagishi and Yamagishi, 1994).

### Results

**FIGURE 3:**
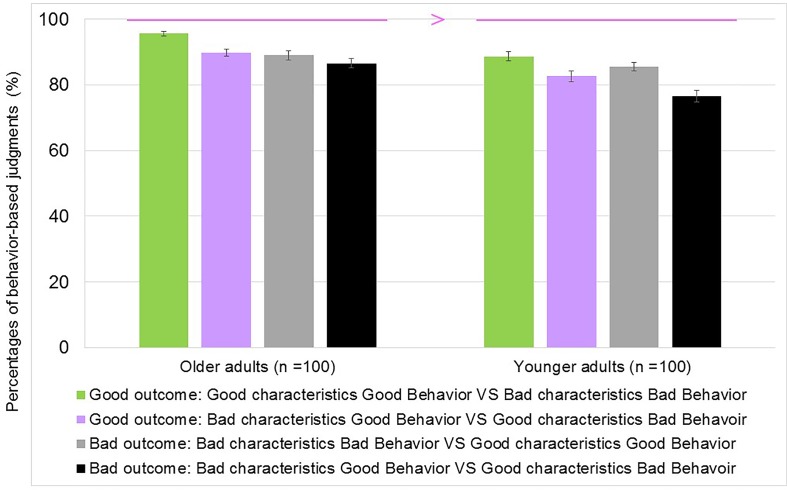
Comparison task: Judgments based on behaviors in Experiment 2. For good outcomes, the green bar shows good characteristics with good behaviors vs. bad characteristics with bad behaviors, and the purple bar shows bad characteristics with good behaviors vs. good characteristics with bad behaviors. For bad outcomes, the gray bar shows bad characteristics with bad behaviors vs. good characteristics with good behaviors, and the black bar shows bad characteristics with good behaviors vs. good characteristics with bad behaviors. Error bars represent 95% confidence intervals.

The percentages of behavior-based judgments are presented in **Figure 3**. For example, in the good outcome condition (“which person is nicer?”), and for the comparison of “good characteristics with bad behavior” and “bad characteristics with good behavior,” the response that a protagonist with “bad characteristics showing good behavior” is nicer than a protagonist with “good characteristics showing bad behavior” is considered to be a behavior-based judgment (in this example, the rater judged the protagonist as a good person based on “good behavior”).

#### Analyses of Behavioral-Based Judgments in the Comparison Task

A group × outcomes × congruencies ANOVA was conducted for behavior-based judgments. The main effect of the group was significant, *F*(1,198) = 10.99, *p* < 0.01, $\eta_{p}^{2}$ = 0.05. The older group made more behavior-based judgments than the younger group. The main effect of the outcomes [*F*(1,198) = 16.96, *p* < 0.001, $\eta_{p}^{2}$ = 0.08] and the main effect of the congruency were also significant [*F*(1,198) = 21.34, *p* < 0.001, $\eta_{p}^{2}$ = 0.10]. However, the interaction between group and outcomes and the interaction between group and congruencies were not significant [*F*(1,198) = 0.02, *p* > 0.05, $\eta_{p}^{2}$ = 0.00, *F*(1,198) = 1.78, *p* > 0.05, $\eta_{p}^{2}$ = 0.01]. Similarly, the interaction between group, outcomes, and the congruencies was also not significant [*F*(1,198) = 3.82, *p* > 0.05, $\eta_{p}^{2}$ = 0.02].

#### Multiple Regression Analyses Based on Behavior-Based Judgments

**Table 2** shows correlations and means of variables in Experiment 2. Multiple regression analyses were conducted to investigate factors related to behavior-based judgments (**Table 3**). We conducted multiple regression analysis to explain the percentages of behavior-based judgments using gender, age, and Trust Scale scores (general trust and caution) as factors^1^.

**Table 2:** Correlations and means (SD) of variables in Experiment 2.

Variables	*M*	*SD*	Behavioral-based judgments	Gender	Age	General trust	Caution
Behavioral-based judgments	0.87	0.2					
Gender	0.5	0.5	0.18^∗^				
Age	44.6	19.1	0.24^∗^	0.01			
General trust	19.3	4.5	0.21^∗^	-0.08	0.43^∗^		
Caution	18.7	4.4	-0.10	-0.02	0.26**^∗^**	0.45**^∗^**	

*N = 200, ^∗^*p* < 0.01, 0.87 for behavioral-based judgments mean that the percentage of judgments based on a protagonists’ behaviors was 87%. Six items for general trust (range = 6–30), 7 items for caution (range = 7–35)*.

**Table 3:** Standardized regression coefficients (*beta* weights) and *R^2^* from the regression analyses based on behavior-based judgments in Experiment 2.

Variables	*Beta*	95% CI	*t*	*p*
Gender (0: Female, 1: Male)	-0.17	[-0.091, -0.012]	-2.59	0.010
Age	0.21	[0.001, 0.003]	2.87	0.005
General trust	0.22	[0.002, 0.012]	2.71	0.007
Caution	-0.26	[-0.014, -0.004]	-3.43	0.001

*R^2^ = 0.13 (*N* = 200, *p* < 0.001). CI, confidence interval for Beta*.

Results indicated that gender (0 for female, 1 for male) was associated with less behavior-based judgments, suggesting that women engaged in behavior-based judgments more than men. Age was also associated with more behavior-based judgments, suggesting that older participants engaged in more behavior-based judgments than younger participants. Moreover, the general trust score was associated with increased behavioral-based judgments, suggesting that people with higher general trust engaged more in behavior-based judgments. Alternatively, the caution score was associated with decreased behavioral-based judgments, suggesting that people with higher caution engaged less in behavior-based judgments.

### Discussion

Results of Experiment 2 indicated that older people made behavior-based judgments more than younger people. Behavioral information is more explicit and observable than information on the characteristics of a protagonist (Komeda et al., 2016). We predicted that older adults would judge the protagonists based on behavioral information, which was supported by the results of the deliberate decision processes examined in Experiment 2.

Moreover, older and younger people engaged in behavioral-based judgments when the outcome was positive more often than when it was negative. Both older and younger people used information on characteristics of the protagonists In the case of negative outcomes more than in the case of positive outcomes. This pattern was consistent with the results of our previous study of typically developing adolescents (Komeda et al., 2016). Moreover, older and younger people engaged in behavioral-based judgments in the case of congruencies (the comparison of “good characteristics with good behavior” and “bad characteristics with bad behavior”) more than in the case of incongruences (the comparison of “bad characteristics with good behavior” and “good characteristics with bad behavior”). That is, both older and younger people used information on the protagonist’s characteristics in incongruent situations more than in congruent situations. This pattern was also consistent with the results of a previous study on typically developing adolescents in daily life situations (Komeda et al., 2016), and in financial decision making situation (Shivapour et al., 2012).

The multiple regression analyses showed that women engaged in more behavior-based judgments than men, and older participants engaged in more behavior-based judgments than younger participants. We predicted that older adults would judge protagonists based on behavioral information. However, we failed to predict that women would make more behavioral-based judgments.

Furthermore, people with more general trust engaged more in making behavior-based judgments, whereas people with more caution engaged less in making such judgments. It is known that general trust is a solution to the problems caused by social uncertainty (Rotter, 1980; Yamagishi and Yamagishi, 1994) and reduce complexity in the environment (Luhmann, 1979, 1988). Thus, people who have a high degree of “general trust” might focus on observable human behavior using a simple perspective rather than focus on changeable characteristics of the situation. On the other hand, the caution scale assesses the extent to which people feel that caution is required for dealing with others. Therefore, people with a high “caution” score might focus on changeable characteristics in more complex situations than merely focusing on observable behaviors.

## General Discussion

Results of Experiment 1 demonstrated that older adults judged protagonists as “good” more often than younger adults. Especially, they judged protagonists to be good in story situations in which these protagonists exhibited good behavior. Experiment 1 used a sentence reading paradigm in which participants could not review previous sentences. In Experiment 2, to control for the accessibility of first (characteristic information) and the second sentences (behavioral-based information), all the sentences in the two stories were presented simultaneously. Under this condition, which gave equal access to characteristic and behavioral information, older adults relied on behavioral information regarding the protagonists, rather than on information about the protagonist’s characteristics.

These results suggest that when rapidly processing information during the social decision making, older adults display biases in deciding that a person is good. These results are consistent with other findings indicating that the age related positivity effect is the result of a top-down motivational shift, promoting emotionally gratifying experiences (Reed and Carstensen, 2012; Neta and Tong, 2016). Additionally, older adults might have judged people based on behavioral information by using a more deliberate decision-making process. Older adults tend to make stereotypical decisions in situations when sufficient time for careful consideration is unavailable (Radvansky et al., 2009, 2010). However, when sufficient time is available for deliberate decision-making, older adults do take behavioral information into account, rather than merely taking information about the protagonist based on stereotypes (von Hippel et al., 2000).

The results of this study indicated no age differences in the ability to use trait-based information about a protagonist’s characteristics when making social judgments, which was consistent with a previous study (Hess and Smith, 2014). Especially, in deliberate decision making, both older and younger people used information about a protagonist’s characteristics when the outcome was bad compared to when the outcome was good. Also, both older and younger people used information on a protagonist’s characteristics in incongruent situations more than in congruent situations.

Importantly, the general trust score was associated with increased behavioral-based judgments, and the caution score was associated with decreased behavioral-based judgments after controlling for gender and the age. Therefore, people with higher general trust engaged more in behavior-based judgments in social decision making. Alternatively, people with higher caution engaged less in behavior-based judgments and engaged more in trait-based information in social decision making.

This study has the following limitations requiring careful interpretation of the results. Firstly, these experiments were conducted using a web-based sample to facilitate collecting data from a sufficient number of older participants. However, experimental research on reading and response times when performing cognitive tasks is necessary. Secondly, no cognitive tasks were examined for assessing the brain function of older people (which is a point that is also related the web-based survey). It is suggested that cognitive assessments should be considered essential in future studies investigating the relationship between the social decision making and aging. Thirdly, stories we created were culture-dependent. It might be difficult for people in other cultures to understand these stories as good or bad. It is suggested that culturally independent material need to be developed to demonstrate the generality of the findings of this study.

The present study focused on the social decision-making processes to clarify why older adults are more likely to become fraud victims. At least, in Japanese society, older people could become victims of fraud even if they do not have dementia (Watanabe et al., 2014). This could be explained by the significant differences in social decision-making between older and younger adults suggested by this study: older people judged others to be good more than younger people, and older people relied on behavioral-based information rather than information on a person’s characteristics, which is surprising, given that both groups were matched for education (as the years of schooling).

In spite of these limitations, however, this is the first study to have investigated social decision making during narrative comprehension using a large sample of older and younger adults. These results are expected to help in the development of programs to prevent older people becoming fraud victims. We hope that our findings would help decrease fraud around the globe.

## Ethics Statement

Electronic informed consent was obtained from all the participants. This study was approved by the Ethics Committees of the Kyoto Prefectural University of Medicine and Keio University School of Medicine. The study was conducted in accordance with the declaration of Helsinki.

## Author Contributions

HK and YE developed the concept of the study. All the authors contributed to the study design of the study. HK and YE performed the data analysis and interpretation under the supervision of TK, JN, and MM. HK drafted the manuscript. YE, TK, YK, JN, and MM provided critical revisions. All the authors approved the final version of the manuscript for submission.

## Conflict of Interest Statement

The authors declare that the research was conducted in the absence of any commercial or financial relationships that could be construed as a potential conflict of interest.

## Footnotes

1 The general trust (6 items, Range = 5–30, a high score indicates more trust) score of the older group (*n* = 21.2) was higher than of the younger group [*n* = 17.3, *F*(1,198) = 46.13, *p* < 0.05]. Moreover, the caution score (7 items, Range = 7–35, high score means high cautious) of the older group (*n* = 19.9) was higher than of the younger group [*n* = 17.6, *F*(1,198) = 14.21, *p* < 0.05].
